# Cancer of the Female Breast—Its Character, Diagnosis, Prognosis and Treatment

**Published:** 1873-10

**Authors:** John C. Peters

**Affiliations:** Pres., in the Chair


*Cancer of the Female Breast—its Character, Diagnosis, Prognosis and Treatment.* Medical Library and Journal Association: Dr. JOHN C. PETERS, Pres., in the Chair.

PROF. WILLARD PARKER remarked as follows:

In the latter part of the last century the man who was particularly connected with the study of this subject, cancerous tumors, was Dr. John Abernethy. Previous to his time, our knowledge upon this subject was in a state of confusion. So completely and successfully did he study this subject of tumors, that, in his time, the results of his investigations were regarded as the *ultimatum*. They were so generally accepted by the profession, that whoever was familiar with what Mr. Abernethy said upon the subject, was supposed to know all that could be known about tumors. These results of the investigations of Mr. Abernethy continued to control the minds of the profession, to a very great extent at least, until perhaps the last twenty-five years. Since that time great changes have taken place, which have grown more particularly out of investigations arising from the use of the microscope and from the researches of the histologists.

The first great departure was introduced by Bichat.

Bichat was the father of general anatomy, and was one of the brightest examples of a thorough scholar that France has ever produced. It was by the influence of his study that the human body was divided into its different systems—the muscular system, the vascular system, the nervous system, etc. In connection with his great work, the study of general anatomy, he took up the study of tumors. In his studies upon that subject, he observed that tumors were growths in the body, abnormal in character, that some of them had a character analogous to the original natural tissues, and that others varied somewhat from the original natural tissues. He observed, therefore, that there were two classes. One *like* the original normal structure, and the other *unlike* the original normal structure, and had a structure *sui generis*.

Bichat died in the year 1802. His pupils, Dupuytren, Andral, Cruvelhier, and others, seized upon this idea which Bichat had advanced, and made the two great classes of tumors with which the profession has been so familiar since their time. They divided tumors into two great classes, one which embraced that class of tumors which were composed of a structure analogous to the natural structures of the body, which they called homologues or homoplasts, and another which embraced that class of tumors which were composed of a structure which found no analogy in the body, which they called heterologues or heteroplasts. They farther observed that the first class were almost always mild in their effects, easily controlled by treatment, and they received the additional name of benign. They also observed that the second class were very apt to destroy life, sometimes very rapidly, were not easily controlled by treatment, and they received the name of malignant. This division of tumors was persisted in for a long time by the French, and it has been in France that the strong advocates of this classification have arisen. In the latter part of the life of Velpeau, that great surgeon began to question this matter of homology and heterology, as the terms had been used in this connection, but his studies and researches assumed no definite shape. The next class of men who attempted to give a solution to the question, What is cancer? were the chemists of France, and of these there were Thenard, Lassaigne, Vauqualin, and others of equal ability. These men made a careful analysis of cancer, with the view of discerning, if possible, something peculiar to cancer: something which perhaps might receive the name of *carcinomatine*, a distinct and fixed substance, the presence or absence of which might always enable us to say whether cancer was present or absent. After a long and careful research, they found that their labors were unsuccessful, and the work was allowed to pass from their hands, and it fell into the hands of the histologists. This class of observers have been diligent in their study, and faithful in looking for some change or modification of the cell-products, shape of cells, or presence of peculiar elements, by which they could distinguish cancer. For a long time it was thought that they had succeeded in making the desired discovery, and the announcement was made that the esential element in cancerous growth was the presence of a certain shaped cell, which was immediately denominated the "cancer-cell." This announcement was very generally accepted by the profession as a solution to the long and vexing problem. It is only a few years since we all had faith that the so-called "cancer-cell," or morphosis, something distinct and definite in its character, which had been found, would enable us to determine whether any given growth was cancerous in its nature, or whether it was not cancerous in its nature. But the truth is, we now know that no one cell at present can be fastened upon as absolutely representing cancerous disease.

The "cancer-cell" theory belonged to the French.

The Germans, on the other hand, never acceded to this proposition, and for some time there was considerable discussion between French and German scholars upon this question. Among the Germans who engaged in this discussion were Virchow, Rokitansky, and others, who have since became pre-eminent in their profession. The study of this subject, however, has been followed up by Virchow particularly, and now we have the result of his investigations formulated and presented to us in his great work upon the pathology of tumors.

First of all he lays down the proposition that everything proceeds from a cell—

"*Omnis cellula e cellula.*"

For a time his opinion was that the embryonic connective-tissue cell had the power of proliferating the cancer-cell, that is, that the connective-tissue cell could produce a cell like itself, and also a cell of a different character, which was the cancerous product. But it now seems to be settled that like only begets like, and the doctrine that the connective tissue is capable of proliferating a cell of a distinct character, which can be called the cancer-cell, is regarded as not being tenable.

The doctrine is now, that the true cancer, so far as we rely upon the cell, is made up of cells which are of the epithelial type.

Failing to discover a peculiar cell by which cancer was to be recognized, both histological and pathological observers have at present settled down upon the point that true cancer is to be recognized by certain peculiarities in its anatomical structure. That is, we have in the first place a stroma made up of connective tissue; that in the interstices of this connective tissue, denominated alveoli, are deposited cells; that these cells are of the large, nucleated variety, and have for their prototype the epithelial cell. The presence of this anatomical arrangement is now regarded as evidence of the presence of true cancer. In other words, that nearly all cancers, if not all, have their origin in epithelial structure. This portion of the subject is one of great interest, and it is to be hoped that, at some future period not far distant, the histologist and pathologist, by aid of the microscope and other means which they have at their command, may be able positively to determine and settle the question as to what changes have taken place in the growths which we now call cancerous. Virchow, pursuing the study still farther, as relating to the clinical history of these growths, has given us several stages.

1. The stage of development or local irritation.
2. The stage of granulation.
3. The stage of differentiation. That is, from being an apparently homogeneous structure, some of the cells go on to make muscular tissue, epithelial structure, etc.

Of these varieties of cells he distinguishes four; that is, cells which are to appear in four different kinds of structure.

1. Connective tissue.
2. Nerve structure.
3. Muscular structure.
4. Epithelium.

Continuing the stages he makes

4. The stage of maturity.
5. The stage of retrograde metamorphosis, or the stage when it has attained its maximum growth and begins to retrograde and die.

This process may take place by ulceration, suppuration, adipose change, and so on.

This embraces all the general remarks I have to make on the question, What is cancer?

Now, with regard to cancer of the female breast. There is hardly any organ in the human body so frequently the seat of this disease as is the female breast. Of course when cancer involves any important organ in the body it becomes the object of great solicitude on the part of the patient and the friends, and on the part of the surgeon a source of great anxiety, and, I am sorry to say, without any satisfactory results attending his efforts to give permanent relief.

In the first place, with regard to its frequency. Upon this point we have some very instructive tables furnished us by a French surgeon, who made his collection of cases during the eleven years immediately subsequent to the year 1830. The whole number of cases he collected, of all kinds, was 9,119.

Of these, 4,222 occurred in organs peculiar to the female sex, and only 48 occurred in organs peculiar to the male sex. This is in the proportion of 88 to 1. The reason for this extreme difference in frequency in the two sexes is not known.

Of the 4,222 cases which occurred in women, 2,996 cases occurred in the uterus; 1,147 in the mammæ; 64 in the ovaries; and 15 in the vagina.

Of the 48 cases which occurred in men, 21 cases occurred in the testes; 10 in the penis; 7 in the scrotum; 5 in the prostate; and 5 in the pap.

A remarkable feature of these observations is that so few cases occurred in the ovaries.

I propose now simply to refer to the cases which have fallen under my own observation. These are cases which I have either treated myself, or have seen in consultation. These observations and notes extend over a period of about 40 years, but the larger portion of these cases have occurred within the last 20 years.

I have upon my tables the record of 295 cases.

In making up these tables I have taken into consideration the following points:

1, date; 2, name; 3, age; 4, civil condition—married, single, or widow; 5, number of children; 6, which breast was involved; 7, family history; 8, personal history; 9, diagnosis, prognosis, treatment; 10, subsequent history.

First, with regard to age:—Average age, $48\frac{7}{10}$ years; oldest, 86 years; youngest, 28 years.

Civil condition:—Married, 190; widows, 53; single, 34; unknown, 18.

Number of children:—Mothers, 186.

Breast:—Right breast, 107; left breast, 130; axilla involved, 71.

It will be noticed that there is a difference of 23 in favor of the right breast. I have also found that the same result has been obtained upon this point by other observers. I can assign no reason why its occurrence should be so much more frequent in the left breast than in the right.

Family history:—Cancer taint, 28; no cancer taint, 208; consumption, 35; consumption and cancer taint, 5; unknown, 36.

It will be noticed here that by far the larger portion occurred in persons who had no history of cancer taint. So far, then, as my tables go, they prove that cancer is not a hereditary disease, which is contrary to the opinion I had always entertained previous to a more thorough examination of the question.

Personal history:—Blows, 38; injury from breastpump, 1; injury from tight dress, 2; inverted nipples, 2; uterine sympathy, 19; mental anxiety, 45; commencing benignant (op. 17), 31; commencing malignant, but living long (op. 14), 15; sloughing and living long (op. 3), 6; cystic (av. age, $57\frac{1}{3}$—oldest, 72, youngest, 33), 9; metastatic, 3.

With regard to the 19 cases which occurred from uterine sympathy, they belonged to that class of cases which formerly were called the irritable tumor of the breast. These cases not uncommonly occur in connection with dysmenorrhœa, and the leading symptoms are swelling and pain in the breast and nipples before menstruation.

I wish to call special attention to the 45 cases marked mental anxiety as a cause for their development. I believe this to be an important point in connection with the development of cancer in the female breast. I believe it is very rare for cancer to present itself where everything passes pleasantly and prosperously in life, and cheerfulness at all times surrounds the individual. I find in many cases, that the development of cancer dates at something which has caused mental anxiety, such as loss of property, loss of friends, long-continued sickness in families, giving rise to great mental solicitude and physical fatigue, etc., etc.

In addition to the above general features, I find that I have the record of 31 cases which began as benign tumors. In this class of cases the tumors had been carried from 10 to 30 years without inconvenience, but suddenly from some cause, mental anxiety being a prominent cause, these tumors commenced to grow rapidly and assume a malignant form. One of these cases I now recall, in whom the tumor had existed from early womanhood. That lady was married when 20 years old, bore two children, her husband was prosperous in life, but suddenly distress in life came from reverse in fortune; and during these dark hours in her history, the tumor took on changes which developed into a rapidly advancing cancerous growth. The tumor was twice removed, but no permanent relief ensued.

Another case was one in which the patient carried the tumor 35 or 40 years without inconvenience, when suddenly the tumor took on new action, and the woman died from the effects of cancerous disease within six years from the time of the manifestation of this new action.

I have another group of cases which began as malignant. They are 15 in number. Of these cases, 14 were operated upon, and remained non-progressive for a long time. These are the cases which have afforded the greatest success in treatment. The following is a condensed statement of the history of these cases subsequent to the operation :—

1. Lived 12 years, and died with cancer of liver.
2. At the end of 27 years was in fair health.
3. Lived several years, and died from accidental injuries.
4. Patient well 22 years after operation. The patient had an issue made in the arm.
5. Lived 36 years, and died of other disease.
6. Patient well 5 years after operation.
7. Patient well 8 years after operation.
8. Patient well 10 years after operation.
9. Patient was operated upon in 1856, disease returned in 1873, but is not active.
10. Living and well 20 years after operation.
11. Living and well 3 years after operation.

12. Lived 7 years after the first operation. This patient had the second operation.

13. Patient living in 1873, 10 years after first seen, and she had no operation.

14. Disease returned 2 years after operation, after great depression of spirits.

I have another group of cases which took on the sloughing process; those in which ulceration and sloughing took place rapidly and continued.

In this group are six cases. Of these, three were operated upon and three were not.

Of the cases in which no operation was performed—

1. Three years' duration, and patient lived 6 years after the sloughing commenced.

2. Nine years' duration, had been ulcerating 3 years, general health unimpaired.

3. Thirty-eight years' duration, and had been sloughing for 10 years.

Of the cases in which operation was performed—

1. Lived 5 years.

2. Operation 12 years ago, patient still living.

3. Patient well 10 years after operation.

This last case was indeed a most remarkable one. When first called to see the lady, I found her in what was apparently an exceedingly unfavorable condition. There was a large sloughing, cancerous mass upon the thorax, which filled the house with the most terribly disgusting odors which emanate from such sloughing masses. The glands in the axilla were not involved. There was no trouble above the clavicle. Operation was resorted to, simply to relieve the patient of the presence of this mass of death. There was no promise whatever of hope in the case. The operation was performed ten years ago, and the patient is still living and in good condition. These sloughing cases, when the surrounding parts are not involved, are to be regarded as favorable cases for an operation, rather than unfavorable. The fact of sloughing should have but little weight in deciding for or against the operation, but if anything, it is to be regarded as favorable.

Another group of cases of which I have taken note belongs to the so-called cystic variety.

Of these cases I have nine. Three of these cases were operated upon, but only one is now known to be living. In that case, cancer taint was present in the family. The patient is now living, ten years after the operation. What is meant by cystic cancer is, that they are tumors containing fluid, and the walls of these cysts or the walls of the tumor contain cancerous structure.

These cases are quite favorable for operation. The common cause for their occurrence is blows.

There is another class which has been denominated metastatic cancer. Properly speaking this term does not express what is understood by these cases. What is understood by the term is, that the disease has ceased its activity in one organ of the body and made its appearance in some other organ of the body. The organs most commonly affected in this way are the uterus and the mammæ.

One case I call to mind, as illustrating this change, occurred in a woman who had cancer of the breast at the age of 54, which subsided in its growth, and at the age of 76 it appeared in the uterus, and was there the immediate cause of the patient's death. I have seen two of these cases in which the transition of the disease was from the breast to the uterus, and one in which the transition was from the uterus to the breast. As a general rule, the constitutional disturbance in these cases is but little, and the patients are generally able to attend to the common duties of life apparently in a very comfortable condition. I will now say a few words with regard to the

FORMS OF CANCER.

Of these we have, speaking particularly of the breast—

*First*—Those beginning as a turbercle in the skin.

*Second*—Those beginning as an ulcer on the nipple. These begin very much as does the epithelial cancer of the lip.

*Third*—Circumscribed. This form occurs more commonly in young persons, usually in fat persons, and is of rapid development. Sometimes this form involves one lobe, sometimes several lobes of the breast, and is something corresponding to the so-called encephaloid form.

*Fourth*—The infiltrated form, or scirrhus. This usually returns very soon after operation, very rapidly involves the lymphatics, and is seen very often in the skin. The disease makes its appearance in the skin in the form of small lenticular masses, sometimes called tubercles in the skin.

*Fifth*—Cystic. This form is usually found in persons past the middle period of life.

*Sixth*—The sloughing variety.

DIAGNOSIS.

Under this head I shall say but very little, for the manner of arriving at a positive diagnosis in these cases you are already familiar with.

I will simply remark, in passing, that the first form, the tubercle, is sometimes mistaken for syphilis. In the skin the appearance is very much like the epithelioma of the lip. The third form is very apt to be mistaken for *fungous hæmatodes.* It is perhaps as near to fungous hæmatodes as occurs in the breast. The fourth form is usually recognized by the pain and retraction of the nipple. The fifth form might raise the question of hydatids of the breast. Hydatids of the breast are exceedingly rare. I have never seen a case. When I find a smooth tumor in the breast filled with fluid, I regard it as being either of the encephaloid or cystic variety of the disease, and the diagnosis between these two is sometimes difficult. The presence or absence of fluctuation and the microscopical evidence are the most reliable.

PROGNOSIS.

*First*—Prognosis is always unfavorable.

*Second*—In tumors commencing benign, prognosis is most favorable.

*Third*—In sloughy tumors, prognosis is more favorable after an operation.

*Fourth*—In cystic tumors, prognosis is more favorable for an operation, and removal of the tumor is advised.

*Fifth*—In tumors which commence malignant, the patients usually die within two years. Sometimes they continue a little longer. Yet of tumors commencing malignant I have 14 cases where the patients lived many years after amputation of the breast.

*Sixth*—Prognosis may be affected somewhat by the method of treatment employed. Hence we have prognosis after compression, electrolysis, caustics and internal treatment, and these will be more particularly spoken of under the head of

TREATMENT.

With regard to treatment, again, I have not much to say. The methods employed may be embraced under the following heads:—

1. Amputation; 2. Caustic applications; 3. Compression; 4. Electrolysis; 5. Medication; 6. Moral treatment.

In the superficial cancer of the breast it is very well to use caustics. The same thing may be said with regard to cancers upon the face. The treatment with caustics in that region is good surgery. When the tumor is situated to any extent below the surface, the idea of caustics is bad surgery.

In two cases which have come under my observation, one died within four days, poisoned by the material used for the caustic application, and the other never reached her home alive.

With regard to the treatment by the use of compression, compressed sponges being usually employed, I have seen no good from it.

With regard to electrolysis, I have seen nothing in it yet to give me any confidence whatever in its use. I have nothing, however, to say against its use. There may be something of value in it, and it should be thoroughly tried. The day was when no knowledge was had with regard to a successful method for the treatment of syphilis, but now we know that by the proper use of proper remedies, that disease can be cured, and charlatanism has left that field almost entirely. With regard to the internal treatment of cancer, I believe very much in it.

### INTERNAL REMEDIES.

I believe that the day must come when something will be accomplished by the aid of internal remedies. Of the remedies now used, arsenic is perhaps the one which commands my confidence more than any other. There is another point in the treatment of cancer which I conceive to be of great importance, and that is, the moral condition of the patient. I believe that it is impossible to cure our patients of cancer unless they are buoyed up by hope. Their surroundings should be of a character that will give them the greatest possible amount of comfort and happiness. Keep them in the sunlight of enjoyment, for darkness is the soil in which cancer flourishes.

### THE QUESTION OF OPERATION.

Now we come to consider an important question: Do we accomplish any good by operations?

There are some who say, Never operate. I think this opinion comes from the older members of the profession, who are inclined to look beyond the simple performance of the operation. The younger men, many of them, say, Operate. Upon this question I perhaps can do no better than to refer you to the opinions of two men who are among the most experienced of the profession, and who have had abundant facilities for making observations. I refer to Paget and Sibley, both of London.

Mr. Paget has shown, from his statistics, that the average length of life after an operation is 43 months, and that the average length of life without an operation is 55 months. Mr. Sibley has shown from his statistics that the average length of life after an operation is 53 months, and that the average length of life without an operation is $32\frac{1}{4}$ months. Here are the results of observations made by two distinguished authorities. I think all that I am justified in saying upon this point is, that every case must be taken by itself, looked at with all its surroundings, before a decision is given either for or against an operation.

The dangers in an operation are not great, if it is decided to perform it.

The following may be regarded as the indications, when attempting to decide upon any given case.

The older the patient, other things being equal, the more favorable for operation. If the cancer has extended so that we have secondary cancer, it is not surgical to operate. Therefore, when the axillary glands are involved, or when the skin is involved, and we have the local and constitutional disease both existing, I regard it as unfavorable for an operation. When the tumor is isolated, and there are no secondary manifestations, the conditions are favorable for an operation, and the sooner it is performed the better is the chance of preserving the life of the patient.

If a patient comes complaining of an irritable tumor of the breast, apparently connected with some disorder of menstruation, I should recommend, first, careful attention to the general health ; and second, if found increasing in size, to remove it at once. There is another condition in which I would operate, and that is in the sloughing cases. Then it is done simply to make the patient more comfortable. Practically speaking, these cases do not belong to secondary cancer, and the operations are not unfavorable. But with all these cases we must use our own discretion. Select the cases, and give them the benefits and advantages of an operation.

Now a few words with regard to the hereditary character of cancer. In the cases which are found in my tables, the cancer taint was present in only 28 of the whole number, 236, whose history upon this point was obtained.

Within the last year I have been examining the Register's Bureau of Statistics in this city, and I find, in a period of time extending over about 70 weeks, there were only 532 deaths from cancer of all kinds and in all organs, while from pulmonary consumption alone there were 6,219 deaths, or as 1 to 11½. When compared with Bright's disease it is found that about three times as many die from that disease as from cancer. From the statistics of the Register's office for the last five years, the following proportion of Americans and foreigners who died of cancer are found :—

Americans, 68 ; foreigners, 154 ; negroes, 5. Savages rarely have the disease.

It would seem as if this disease of the breast is found in certain conditions of life, and that in these conditions it is upon the increase.

Without pursuing the discussion of this subject farther, I will close by saying, that the conclusions to which I have arrived are chiefly as follows :—

1. That the disease is not hereditary, or if so, only in a very limited degree.

2. That the disease begins as a local disease positively and purely. It becomes constitutional, just as syphilis begins a local disease and becomes constitutional.

3. That the disease occurs in those of vigorous health, instead of being connected with those conditions in which consumption occurs.

4. That cancerous parents may beget tuberculous offspring. That is, feeble constitutions arising from the effects of cancer will not beget cancer, but the diseases which follow in their line are tuberculous.

5. That the moral condition has a powerful influence on the development or the prevention of the development of cancer.

6. I am very forcibly struck by the parallelism and analogy existing between cancer and syphilis. Both begin by local irritation. Syphilis is inoculable, but cancer has not been proven so to be. In this respect they differ from each other. We have secondary syphilis, and we have secondary cancer. We have tertiary syphilis but perhaps it cannot be said that we have tertiary cancer, unless it can be said that cancer is tertiary when it affects the bones, as it sometimes does.

In conclusion, I have to say, that we must not give this subject over as an unprofitable one for study and observation. Many diseases have run rampant which finally have been made to yield to treatment, and we may hope that the same thing may yet be accomplished with reference to cancer. The work of the histologist and pathologist may yet bring us into the light, and the day may come when we can say of cancer, as we now can say of syphilis, It can be cured.

Dr. Fordyce Barker: Mr. President, my apology for departing from my usual rule with regard to surgical questions and operations is, that I may perhaps suggest some new fields for inquiry and observation, and perhaps bring out some new ideas in the course of the discussion by these suggestions. In regard to surgery, I am no expert. I do not pretend even to interfere with it, and it is therefore somewhat embarrassing to speak upon a subject which really belongs to the surgical department. I have, however, had occasion to study the subject of cancer with great interest, and perhaps with a large experience, and have, therefore, for many years taken every pains to inform myself with regard to the progress of science, and have felt an interest in its bearing upon the question of its manifestation in the form in which it occurs secondarily, which in its most frequent form is in that of cancer of the breast.

In alluding to certain points in connection with the general subject, I will refer to one or two cases in connection with my own personal experience. Previous to my coming to this city, I was obliged to practice more or less in general surgery, and in the course of that time I was called upon to amputate the breast thirteen times, for what I supposed to be cancer of that organ. I have listened to the statistics from the gentleman who has already occupied your attention, with great interest and with great pleasure, because, in almost every point, while they have not corresponded with published statistics as we now have them, they have corresponded with my own. In four of these thirteen cases in which I operated for cancer of the breast, I know nothing of the results. Two of the thirteen cases are still living. All of the seven remaining cases died at periods varying from eighteen months to four years after the operation. A curious point in relation to them was, that the one who lived the longest—and this point I have not seen alluded to by any author—was the patient who was the oldest. That patient was 71 years of age when I operated, and had been afflicted with the disease some four or five months when I first saw her.

There was no apparent return of the disease until several months afterwards, and then there was probably a return of the disease to some internal organ. The point is this: whether the progress of pathological changes is not exactly in the same ratio as the metamorphosis of tissue in relation to age; whether in persons of advanced life we may not account in this way for the longer exemption from a fatal termination of the disease than when the disease occurs in those who are less advanced in age.

In 1858, although I had refused to have anything to do with general surgery, and confined my operations entirely to the obstetrical department, I had one patient who absolutely refused to permit any one else to operate upon her except myself. I accordingly removed her breast. The axillary glands were not involved, but the disease returned within a very few months, and the patient died eleven months after the operation.

The second case which I will refer to is a rather curious and rather exceptional one. It occurred in the year 1860, in a lady 43 years of age, and she had had the disease for several months when I first saw her, and in what I regarded as a very malignant form. That person, again, utterly refused to have an operation performed unless I would perform it myself, and I accordingly performed the operation, assisted by Dr. Foster Swift and Dr. Charles Phelps. In that case acupressure was employed, as I believe, for the first time in this city, and I was very much interested and pleased with the effect of acupressure in diminishing the amount of suppuration, which in that case was very slight indeed. That patient was operated upon in April, 1860. In my own belief, and in the belief of the microscopist, it was one of the most malignant forms of this disease of the breast, and yet the woman was alive in 1871. I simply mention this case as a small contribution to the number of successful operations, in the sense of curative, in cases of carcinoma of the breast. That specimen was afterwards presented at the New York Pathological Society, and the minutes of the meeting, which were published in the *Medical Record*, represented it as being presented by Dr. Swift and that the operation had been performed by Dr. Parker, which is a fair illustration of the uncertainty of surgical glory. With regard to statistics in determining whether a surgical operation shall be performed or not, most modern writers agree that operations do, in a certain proportion of cases which are judiciously selected, absolutely and positively prolong life, relieve suffering, and in some cases actually save life. The diametrical opposition which the statistics of some surgeons have to those of other surgeons who are equally well situated for making observations, may perhaps be explained in this way. One surgeon may be of the opinion that the disease is, primarily, always a local disease, and that its constitutional character is secondary to the local disease, which manifests itself differently in different cases. If this theory be correct, the proper method of treatment is the early extirpation of all suspicious-looking growths. On the other hand, other surgeons are of the opinion that the disease is a constitutional disease; that operations are deleterious in their effects, and should not be resorted to until all other means have failed to arrest its progress.

Again, some surgeons who have a greater fondness for operations than others, will remove a suspicious-looking growth much earlier than those surgeons who are less fond of operations, so that in some cases it may be that the delay in the performance of the operation has permitted the disease to make such extensive ravages upon the general system, that the operation, if performed at all, can only be performed with the expectation of giving some relief from distressing symptoms.

I began in early life as a most enthusiastic believer in the numerical system, regarding it as a most efficient means for advancing our knowledge of disease. But my experience has proven to me that statistics which ordinarily receive publication are extremely unreliable, and that they form a most unstable foundation upon which to predicate future action, whether it shall be for the formation of an opinion or made the basis of an operation. The statistics which the author of the paper has given us relative to the comparative frequency of cancer of the breast singularly accord with the statistics taken from the cancer hospitals in the city of London. Out of 7,800 cases which were under treatment in that city between the years 1851 and 1861, 4,388 were cancer of the breast. This is from an entirely different sphere of observation, and yet the result of the observation shows that the female breast is one of the most favorite places in the human body for the development of this disease. It seems to have an elective affinity for the female breast, and perhaps in the progress of etiology and the science of physiology the reason for this elective affinity will be discovered.

The next point which I will notice in connection with the paper, is with regard to hereditary predisposition to the disease. I feel quite confident that I should never have read a paper which I did read, and which was published by the Academy of Medicine, upon "The Clinical Study of Cancer of the Uterus," had I not been thoroughly convinced upon this point. When I came to study my own observations, I found that some of them were so different from the published statements in published works that I felt doubtful about reading them without consultation with some of my personal friends. My own statistics with regard to hereditary predisposition to cancer of the uterus almost exactly correspond to the observations of the author of this paper with regard to hereditary predisposition to cancer of the female breast.

Another very interesting point to me was, that the author of the paper has found so much larger proportion of cases of cancer of the breast where hereditary predisposition to cancer was entirely absent, but where hereditary predisposition to tubercles was present. The results of his observations upon this point give the same relations which are found in my own statistics, and I believe that the idea of hereditary predisposition to cancer should be denounced, and that this denunciation should be pronounced boldly by physicians.

There were a few points to which no allusions were made, and concerning which I wish to make some inquiry.

What is meant by a cancerous cachexia? In my earlier experience I was always looking for something like a cancerous cachexia, but my later experience and observation have taught me to become a non-believer, and I do not now believe at all in cancerous cachexia, as the term is commonly used. I have seen patients in the most advanced stages of cancer of the uterus, and in almost all its various phases, when they presented the appearance of robust health. The cachexia, when it does appear, is to my mind not a measure of the influence which has been produced by the simple presence of cancer in the system, but rather from associated lesions of the various organs of the body.

These are my observations with regard to cancer of the uterus, and I should like to know whether the same thing has been observed with regard to cancer of the breast.

Another point, which was not alluded to, and concerning which I should be pleased to gain some information, is, with regard to the value of pain as a symptom in cancer. I am of the opinion that it is a symptom of uncertain value in aiding us in determining the existence or non-existence of cancer of the uterus. I have seen patients in the advanced stages of the disease without the slightest suspicion having been raised with reference to the presence of the disease by any pain. My own opinion is, that pain is simply a measure of the influence which the disease has had upon the contiguous and adjacent tissues. Cancer may occur so as to interfere with the functions of the uterus, or affect the subperitoneal tissues; and when these tissues are affected we are sure to have pain, and in some of these cases the pain is most atrocious. In other cases, where the disease presents more malignancy, the pain is sometimes very trivial. Whether the amount of pain is in relation to the amount of influence which the disease has upon the adjacent and contiguous tissues, I am not able to say, but simply throw it out as a question for consideration.

From time immemorial there has been an attempt made to destroy cancer by the use of every variety of known caustics. It has been a favorite resort of empiricism, and the most successful and perhaps the most lucrative of all charlatanism has been seen in the use of caustic agents to destroy the local manifestations of cancer. As a consequence of this, of course, a great majority of the surgical world have been satisfied with regard to the uselessness of such attempts. My own prejudices have always been against this method of treatment. I once attempted to make some observations respecting this plan of treatment as it was then adopted in St. Bartholomew's Hospital, and the whole process was so revolting that I did not pursue my investigations farther, and the result of my observations was not at all favorable.

In the year 1870, however, I was consulted by a lady who had a tumor in the breast which was very suspicious in its character, and which I watched for some weeks, when I regarded it as cancer, and urged upon my patient the importance of having it removed at once. But that patient utterly refused to have any cutting operation performed. At that time I had been studying up the subject somewhat, and among other works which I had read was Marsden's work upon the use of caustics in the treatment of cancer.

The same summer, while abroad, I visited the hospital in which Dr. Marsden had made his observations and applied his treatment, and saw the results of this treatment. I became so much interested in this plan of treatment and was so highly pleased with it, that, upon my return, I recommended to my patient to submit to the treatment by the use of caustics. After some delay she consented. The form of cancer from which she was suffering was apparently of the most malignant type, and at the time I commenced the treatment the mass was about two inches in diameter, which is the extreme limit in size permissible to be treated by this method. In the course of eighteen days after the first application was made, the mass came away, the process of cicatrization was completed in a short time, and there has not been the slightest appearance of return up to this time.

Another case to which I wish to make reference, was in a patient who had had two sisters die with cancer of the breast, but her father and mother were still living at the time she consulted me. Not the slightest suspicion of cancer could be traced in either member of the family. One sister died some six or seven years ago from cancer of the breast. The other sister I was called to visit, and I found the axillary glands involved in the disease; there were evidences of what is known as the cancerous cachexia, and I called for council. Dr. Van Buren was called in consultation, but the case was regarded as utterly hopeless, and the patient died without an operation.

The third sister came under my observation for epithelioma of the uterus. That patient I operated upon in 1866, removing the cervix uteri by amputation. It is now seven years since the operation was performed, and she remains in the most perfect health.

About five years ago a lady consulted me with regard to a suspicious-looking tumor in her right breast. She was under my observation for about two years, and received treatment, but I never was of the opinion that the growth was malignant. At the end of two years it entirely disappeared. In February, 1873, that patient came back to me with a tumor in her left breast, which I regarded as true cancer of the breast. The tumor had been observed for more than a year, and when I saw it, the nature of the case seemed clear and positive. Its removal was recommended. Consultation was held, to satisfy the patient with regard to its nature, the propriety of its removal, and if decided to remove it, how it should be removed. It was decided to remove the tumor by Marsden's treatment, and the treatment was accordingly commenced upon the first day of April. The amount of pain which the patient has suffered during the course of the treatment has been very insignificant indeed. She has been up most of the time, has been able to be out riding some of the time, and it is now eighteen days since the first application, and the slough is just ready to come away. The treatment of this case thus far has been very pleasant. What the result of the case may be it is impossible at present to decide.

I will now describe the plan of treatment as given by Dr. Marsden—the plan which he professes to have derived great success from, not only in a very considerable number of cases of cancer of the breast, but in the treatment of cancer of various parts of the body, and even of cancer of the neck of the uterus.

This method of treatment is limited to cases in which the surface of the tumor does not extend over two inches. Care must be taken that the paste is of sufficient consistence so as not to flow beyond the point to which it is applied. The general formula for the preparation of the caustic is to combine arsenious acid and mucilage in such quantities as to make a thick paste, and the formula commonly employed for this purpose is—

| | | |
|---|---|---|
| R. | Arsenious Acid, - - - - - - | dr. ij. |
| | Mucilage, - - - - - - - | dr. j. |

This paste is spread over the surface of the tumor, and two or three layers of lint spread over that. The lint absorbs all the surplus paste and protects from farther cauterization. The first application is left on for twenty-four or forty-eight hours, according to the extent of surface, and then removed by gently soaking it with warm water. After the old paste has been removed in this way, one judges from the impression made with regard to a farther application of the caustic. These applications are to be continued until a line of demarcation entirely surrounding the diseased structure is shown. Then the lint is soaked and removed, and a bread-and-water poultice applied, and changed every few hours. At first there is sometimes considerable inflammatory action set up, but the amount of pain is very inconsiderable as compared with the use of the knife, and the process of cicatrization is equally painless and satisfactory.

The shock to the system, as a rule, is very much less. The constitutional effect of the arsenic in this case was very slight, lasting only a few hours, and then passed away. Indeed, the moderate constitutional effect of arsenic I have long believed to have a certain positiveness in the treatment of cancer, in that it retards the proliferation of cancerous tissue. I mention these cases with the hope that it may contribute something to our knowledge of means by which we may meet this most terrific disease.

Dr. Sands: Mr. President, I shall merely call the attention of the Association to a few points in connection with the nature of cancer, the classification of cancer, the etiology of cancer, and the treatment of cancer. I am more and more convinced, until surgeons can have the aid of the pathologists, until they can work together in the investigation of malignant growths, that but little advance can be made with regard to the important subject of the therapeutics of cancer.

It is a fact with which we are all familiar, that at the present time various kinds of tumors are removed from the body, and the most varying opinions are expressed with regard to these cases. It is not long since that I removed a female breast, and one surgeon who was present expressed the opinion that it did not need any microscope to enable him to determine that it was cancerous; but a microscopical examination proved it to be a case of pure adenoma of the breast. I think that most surgeons, as well as pathologists, are willing to give an anatomical rather than clinical meaning to the term cancer. The term cancer can no longer be considered synonymous with malignancy. All cancerous tumors are malignant, it is true; but, on the other hand, there are many tumors which are malignant in their nature, to which the term cancer cannot be applied, because they have not the anatomy of the tumors described under that term. Among these we have the sarcomata, myxomata, epitheliomata, lymphomata, and some others. If we are to make any progress in our investigation of this subject of tumors, each tumor should be examined immediately, with the view of determining the true anatomical elements of which it is made up.

With regard to the etiology of cancer, of course it is very interesting and important to determine whether cancer is, or is not, a local disease, whether the condition of the patient is always consequent upon a constitutional taint, or whether it is simply a symptom incident to a local morbid state. Different views have already been given upon this point; and in favor of its being a local disease primarily, there is a degree of plausibility, when we go a certain distance. The occurrence of cancer secondarily can be explained in this way, when it occurs in or near the original seat of the disease. But it does not so easily explain the occurrence of cancer in parts *remote* from the original seat of the disease. In illustration of this point is the following case:

I had a patient under observation who was suffering from primary melanotic cancer of the lymphatic glands in the axilla. The diseased glands were removed, and the patient had a good recovery, but afterwards suffered from secondary cancer of the scalp. In the latter situation the disease was identical with that of the lymphatic glands in the axilla.

Another case illustrating the same point occurred in a person from whom I removed, about eighteen months ago, a small epithelioma of the face. Dr. Parker had removed a similiar growth from the same situation before. This gentleman made a good recovery, but he is now suffering from epithelioma of the rectum, and is rapidly declining in health. I have still another case, where it is yet more difficult to explain the secondary occurrence of the disease upon the theory that cancerous tumors are transplanted. That case was seen in St. Luke's Hospital, and was a small tumor which occurred in the left ventricle of the larynx. The tumor was removed, and the patient made a good recovery. The disease was never reproduced in the original locality. The nature of the tumor was determined by microscopical examination to be cancerous. About eighteen months subsequent to the operation, the patient again entered the same hospital, and died twenty-two months after the original operation. When post-mortem was made, cancerous disease was found in the suprarenal capsules of both sides and one ureter. It would seem hardly possible to explain this case upon the theory that secondary cancers are always the result of primary cancers; and all these cases which I have mentioned go very much to show that cancer is a constitutional and not a local disease. Some surgeons go so far as to say that all tumors are expressions of constitutional disease. It is a well-known fact that, in some cases, benign tumors are so numerous that their occurrence in such numbers can only be explained upon the theory that they are dependent upon some constitutional cause.

With regard to hereditary tendency of cancer, Dr. Parker's statistics are very gratifying; but there are some facts which I think show that in some cases, at least, cancer can be inherited. Cases like the following would seem to indicate that the disease has a hereditary tendency. In that case autopsy demonstrated that the gentleman died from cancer of the stomach, and not long afterwards a son of the same gentleman died with cancer of the same organ. I have already in mind several cases equally well marked, which it seems to me clearly indicate that the disease has a hereditary tendency. With regard to the treatment of the disease, I have had no experience with reference to the use of caustics. I have, however, a very decided conviction that the knife is the remedy, so far as there is a remedy in this disease. I do not believe that cancer is purely a local disease, yet if the knife can be applied early, and remove not only all the tissue of the disease, but the tissues about the disease, there may be a chance for a long interval before a return of the disease, in some cases.

The reasons why I would advocate the treatment of cancer by operation with the knife are chiefly threefold. One is, that the nature of the tumor cannot be determined prior to its removal. Another reason is, that it now and then happens that a tumor of undoubted malignancy is cured by this surgical operation. Finally, I think an operation is justifiable often upon the ground of expediency. The chances are increased, if the patients die from cancerous disease, that they will die not from the external progress of the cancer, but that they will die from cancer of some of the internal organs. It is very well known that the return of the disease is very often upon the internal organs after the external disease has been removed, especially upon the lungs, and a death from cancer of the internal organs is not so painful and not absolutely loathsome, as when permitted to pursue its ordinary course externally.

Dr. Buck related the history of a case of cancer cured in the person of a lady about 40 years of age. The tumor was in the breast, and possessed all the characteristics of scirrhus. It was removed, and more than 30 years after the operation it was ascertained that the patient was yet living and in comfortable health.

Another creditable case was in a lady who was very timid and despondent. One determining point in this case was, to perform an operation in order to infuse hope into the mind of the patient with regard to her recovery. She did make a favorable recovery, and subsequently returned to France. That lady was well for two years, and I was fully convinced that she would not have survived as long as that without the operation. In these days of anæsthetics we may more readily advise an operation, for the patients are entitled to the respite of hope and comparative comfort which may be more than an equivalent for the fears and sufferings which they may experience without the operation.

With regard to operating for the removal of cancerous breasts in very corpulent persons, my experience has been very discouraging. I always dislike to perform an operation upon such patients, for in some cases which I have seen their life is drained away, as it were, from the wound.

Dr. Thompson: Mr. President, upon perusing the records of the New York Pathological Society, from 1845 to the present time, I find that 55 cases of carcinoma of the stomach have been reported by various surgeons, of which Dr. Parker reported four. The first case was reported by the President of this Association, Dec. 24, 1845.

Of those 55 cases, 23 were males and 18 females.

In fourteen cases the age of the patient was not reported. The average age of the cases reported was $49\frac{1}{3}$ years, which is a little above the average age of Dr. Parker's cases, which he reports at $48\frac{7}{10}$ years.

*Remarks by the President.*—Gentlemen and Associates: We may well congratulate ourselves upon the results of this evening's investigations and discussion. We have become better acquainted with the real nature of cancer of the breast, and with those hygienic, therapeutic and operative means which will not only delay its progress and mitigate its dire effects, but which may in a certain number of cases remove it entirely.

The time has now arrived when its treatment is removed from all empiricism, and the regular profession is in possession of means which will accomplish vastly more for the sufferer than any boasted specifics, or any irregular or mysterious practice.

We now know that cancer of the breast is generally a simple and local, not a constitutional or hereditary affection. It is a mere excessive growth and proliferation of normal epithelium. The first change is a large accumulation of epithelium within the ducts of the gland, just as in ordinary adenoma. But soon the walls of the ducts are not only invaded by epithelium, but this is also found outside the ducts amongst the inter- and peri-glandular connective tissues, which in their turn are thrown into a state of active proliferation, and become infiltrated with small round cells. Now, we have every reason to believe, from the vast experience of Weeden Cooke, that the muriate tincture of iron and other mineral astringents will arrest these processes in their incipiency, and much delay them in their advanced stages.

We also know that the epithelial cancer-cells are not cemented together by any intercellular substance, and that the lymphatics communicate with the alveoli in which the loose and detached cells are contained. The mineral astringents will prevent the absorption of the infecting cells into the lymphatics, and the further contamination of the system.

If the disease be farther advanced, we have every reason to believe that Marsden's and Cooke's pastes will accomplish more than any caustic vaunted by quacks.

Finally, with proper precautions, it has been proved that the knife may often be used very successfully. There is an active and passive stage of cancer; and when the tumor is removed in its active condition the constitution resents this premature interference, and the disease returns rapidly after the operation. The local soothing measures recommended by Weeden Cooke should be used until the tumor has ceased to grow, and then exsection may be performed with safety.—*New York Med. Record.*

